# Correction: Kazmi et al. Formulation and Evaluation of Kaempferol Loaded Nanoparticles against Experimentally Induced Hepatocellular Carcinoma: In Vitro and In Vivo Studies. *Pharmaceutics* 2021, *13*, 2086

**DOI:** 10.3390/pharmaceutics14040882

**Published:** 2022-04-18

**Authors:** Imran Kazmi, Fahad A. Al-Abbasi, Muhammad Afzal, Hisham N. Altayb, Muhammad Shahid Nadeem, Gaurav Gupta

**Affiliations:** 1 Department of Biochemistry, Faculty of Science, King Abdulaziz University, Jeddah 21589, Saudi Arabia; fabbasi@kau.edu.sa (F.A.A.-A.); hdemmahom@kau.edu.sa (H.N.A.); mhalim@kau.edu.sa (M.S.N.); 2 Department of Pharmacology, College of Pharmacy, Jouf University, Sakaka 72341, Saudi Arabia; afzalgufran@ju.edu.sa; 3 School of Pharmacy, Suresh Gyan Vihar University, Jaipur 302017, India; drgaurav.gupta@mygyanvihar.com; 4 Department of Pharmacology, Saveetha Dental College, Saveetha University, Chennai 600077, India

In the original publication [1], there was a mistake in Figure 2 as published. The image of Figure 2 was not clear. The corrected Figure 2 appears below. The authors apologize for any inconvenience caused and state that the scientific conclusions are unaffected. The original publication has also been updated.

## Figures and Tables

**Figure 2:**
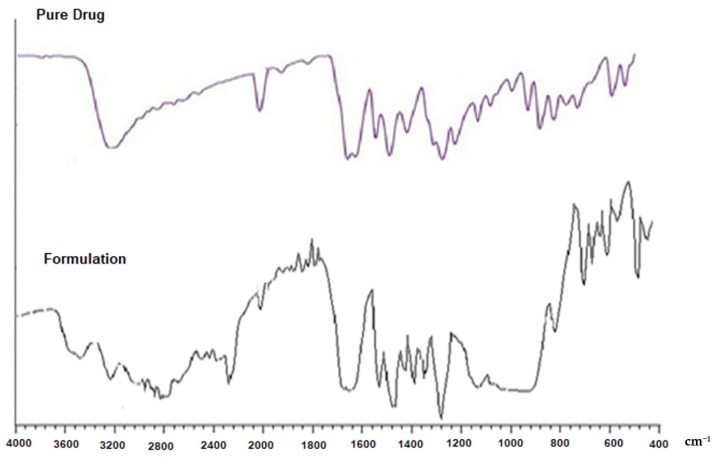
FTIR spectra of the Kaempferol (pure drug) and the prepared Kaempferol nanoparticle.
